# Outlier analysis: Natural resources and immigration policy

**DOI:** 10.1371/journal.pone.0261533

**Published:** 2022-01-13

**Authors:** Seung-Whan Choi

**Affiliations:** Department of Political Science, University of Illinois, Chicago, Illinois, United States of America; Istanbul Medeniyet University: Istanbul Medeniyet Universitesi, TURKEY

## Abstract

This replication underlines the importance of outlier diagnostics since many researchers have long neglected influential observations in OLS regression analysis. In his article, entitled “Primary Resources, Secondary Labor,” Shin finds that advanced democracies with increased natural resource wealth, particularly from oil and natural gas production, are more likely to restrict low-skill immigration policy. By performing outlier diagnostics, this replication shows that Shin’s findings are a statistical artifact. When one outlying country, Norway, is removed from the sample data, I observe almost no significant and negative relationship between oil wealth and immigration policy. When two outlying countries are excluded, the effect of oil wealth completely disappears. Robust regression analysis, a widely used remedial method for outlier problems, confirms the results of my outlier diagnostics.

## Introduction

Marcus Cicero, the famous Roman political theorist, once said, “by doubting we arrive at the truth.” In the spirit of Marcus Cicero, contemporary political scientists question influential theories and empirical findings that have been widely accepted in the discipline of political science. Gary King, the famous Harvard professor, is the pioneer in the area of scientific inquiry. He underscores that “the most common and scientifically productive method of building on existing research is to replicate an existing finding–to follow the precise path taken by a previous researcher, and then improve on the data or methodology in one way or another” [1 page 445]. Replication studies appeared in prestigious outlets include Ashworth et al. [2], Choi and Samy [3], Crabtree and Golder [4], Imai, King, and Rivera [5], Jackman [6], Clarke and Stone [7], Park and Colaresi [8], Powell and Tucker [9], and Zigerell [10]. These replication studies brought about some controversy; nevertheless, they have significantly contributed to increasing empirical rigor in each research program. Put differently, empirical political research is not immune to errors of negligence and, therefore, replication studies remain necessary for continued scientific progress.

This replication features the importance of outlier issues related to OLS regression analysis. Unfortunately, many students of international relations have long neglected influential observations in their data analyses. A notable exception is Cederman and Girardin [11] and Fearon, Kasara, and Laitin [12] who debated the impact of outlier cases on the relationship between ethnic minority rule and civil war onset [see also 13,14]. To illustrate the significance of outliers, I introduce a recent empirical study whose main findings are overturned once a few outliers are removed from the sample data. In his article, “Primary Resources, Secondary Labor,” Shin [15] argues that natural resource-rich (from now on “resource-rich”) democracies, particularly those with high revenue from oil and natural gas production, are more likely to restrict low-skill immigration policy. For empirical testing, Shin puts forth two hypotheses.

The first hypothesis stipulates that the higher oil income, the more likely a country is to implement strict immigration policy. Resource-rich democracies create a high demand for capital but a low demand for labor-intensive jobs in which immigrant workers are heavily employed. Since resource-rich democracies no longer need a great deal of immigrants to run outgoing labor-intensive industries, they have little to no incentive to liberalize immigration policy. In other words, Shin’s study looks into firm preferences on immigration policy among developed democracies. He contends that when firms move from labor-intensive to capital-intensive businesses thanks to oil booms, they are less likely to support open immigration policy and more likely to side with anti-immigration movements. Consequently, resource-rich democracies are less likely to welcome immigrant workers, leading to heavier immigration restrictions. The second hypothesis states that immigration policy becomes more restrictive when oil income interacts with trade liberalization. This hypothesis assumes that the degree of trade openness conditions the extent to which oil income reduces liberal immigration policy. Interestingly, since Philips Petroleum discovered petroleum resources in 1969, Norway has gradually expanded trade liberalization and benefited from high prices of petroleum. “Unlike many other countries rich in raw materials, natural resources have helped make Norway one of the most prosperous economies in the world” [16]. During this economic expansion, “Norway’s oil boom led to the decline of labor-intensive industrial sectors–a necessary condition for the 1975 immigration ban” [15 page 815].

Based on a newly expanded dataset on immigration policy across 24 wealthy democracies during the period from 1801 to 2013, Shin’s empirical analysis finds supporting evidence that oil-rich democracies are more likely to restrict low-skill immigration policy, especially when faced with competition from foreign firms in the international trade market. His empirical tests are complemented by an in-depth case study of Norway in which a booming oil industry induced strict immigration policy, thereby driving away inflows of immigrant workers. Shin’s study should be commended for presenting a mixed-method strategy that combines both advanced statistical techniques and an intensive case-study analysis.

Shin’s study relies on ordinary least squares (OLS) as an estimation method since the dependent variable, the level of immigration policy, is continuous. For OLS regression analysis, researchers routinely conduct outlier diagnostics to ensure the robustness of their findings. When abnormal observations are not addressed with care, one may end up with erroneous inferences and conclusions because they may drastically change the magnitude of regression coefficients and even the direction of coefficient signs (i.e., from negative to positive or vice versa) [13,14,17,18]. Gujarati [19 page 540] defines an outlier “as an observation with a large residual”–a larger vertical distance between the actual observation and the predicted regression line than is generally true for the rest of the data. Such an observation may have high leverage if it is disproportionately farther away from the rest of the data points. A high leverage observation may also be influential, which means that it pulls the regression line toward itself. The bottom line is, the more extreme the outlier problem is, the more biased the regression estimates are.

Although there are multiple methods for detecting outliers, I employ two commonly used graphic diagnostics for analytical simplicity: residual-versus-fitted plot and leverage-versus-squared-residual plot. The rationale for the use of graphic analysis reflects the old saying, “seeing is believing.” When I perform graphic outlier diagnostics on the OLS regression models appeared in Shin’s study, I notice flaws in his empirical findings and validity issues with his case selection of Norway that is an outlier rather than a typical oil-rich democracy. And then, I show almost no significant and negative relationship between oil wealth and immigration policy when Norway, the most outlying case, is removed from Shin’s sample data. This finding, to a certain extent, echoes Jackman’s [6] research in which the presence of Norway (with its North Sea oil) bolsters the association between leftist governments and economic growth in the 1974–80 period. When two outlying countries are dropped from Shin’s sample, the negative effect of oil wealth completely disappears. Robust regression analysis, a widely used remedial measure for outlier problems, confirms the results of my outlier diagnostics. Therefore, my outlier diagnostics in this replication provide evidence that Shin’s findings are a statistical artifact in that oil wealth has no bearing on immigration policy in developed democracies.

## Replications

As the first step of outlier diagnostics, I explain how Shin’s study builds statistical models and why I choose to replicate four of its OLS regression models that appeared in Tables 3 and 5 [15 pages 810 and 813].

Shin’s study designs two statistical models. To test the first hypothesis–increased oil wealth restricts liberal immigration policy, he introduces the following:

Immigration Policy_*it*_ = f (Immigration Policy_*it-1*_, Natural Log of Oil Income Per Capita_*it-1*_, Control Variables_*it-1*_)

Since the dependent variable is continuous, the study chooses OLS with panel-corrected standard errors as the estimation method. To avoid potential endogeneity and reverse causality, all the independent variables in the model are lagged by one year. The statistical model also takes into account time and country fixed effects as well as country-specific time trends.

To test the second hypothesis–increased openness for international trade heightens the degree to which increased oil wealth reduces immigration-policy openness, he builds a multiplicative regression model as follows:

Immigration Policy_*it*_ = f (Immigration Policy_*it-1*_, Natural Log of Oil Income Per Capita_*it-1*_, Aggregate Tariff Rate _*it-1*_, Natural Log of Oil Income Per Capita_*it-1*_ * Aggregate Tariff Rate _*it-1*_, Control Variables_*it-1*_)

The second model’s specification is the same as the first one except for the presence of interaction-related terms: Natural Log of Oil Income Per Capita_*it-1*_, Aggregate Tariff Rate _*it-1*_, and Natural Log of Oil Income Per Capita_*it-1*_ * Aggregate Tariff Rate _*it-1*_. Aggregate Tariff Rate _*it-1*_ is introduced as a proxy measure for international trade openness.

I choose to replicate Models 1 and 6 of Table 3 (p. 810) and Models 19 and 21 of Table 5, which appear in Shin’s [15] original study on page 813. These four models are representative of the core findings of Shin’s empirical analysis. I successfully replicate the results with no difficulty thanks to the replication materials posted by Shin on his website (https://www.adrianshin.com/research.html). The replication materials are well-organized, including Stata data and do-files. In Table 1, I report my replicated estimates that are exactly the same as Shin’s study. Ln(oil income per capita)_*t-*1_ is statistically significant and in the hypothesized direction (negative) across the board, while Tariff rate_*t*-1_ and Ln(oil income pc) × tariff_*t*-1_ are (in)significant with a positive sign. These results verify the two main arguments of Shin’s study: (1) oil wealth is detrimental to liberal immigration policy; and (2) oil wealth and trade tariff together cause advanced democracies to implement restrictive immigration policy.

**Table 1:** Oil wealth and immigration policy: Replication.

* *	1	2	3	4
* *	1801–2013	1946–2013	1951–2013	1961–2012
* *	Shin’s Model 1	Shin’s Model 6	Shin’s Model 19	Shin’s Model 21
Immigration policy_*t*-1_	0.920***	0.904***	0.887***	0.862***
	(0.006)	(0.007)	(0.014)	(0.016)
Ln(oil income per capita)_*t*-1_	-0.006**	-0.011***	-0.023***	-0.034***
	(0.002)	(0.003)	(0.006)	(0.007)
Tariff rate_*t*-1_			0.005	0.002
			(0.003)	(0.005)
Ln(oil income pc) × tariff_*t*-1_			0.002*	0.002*
			(0.001)	(0.001)
Ln(GDP per capita)_*t*-1_		-0.035	-0.046	-0.061
		(0.027)	(0.046)	(0.080)
GDP growth_*t*-1_		0.064	-0.203	-0.146
		(0.072)	(0.169)	(0.205)
Ln(population)_*t*-1_		0.103	-0.201	-0.8756**
		(0.112)	(0.235)	(0.285)
Polity score_*t*-1_		-0.008*	-0.009	-0.043*
		(0.003)	(0.008)	(0.018)
Real effective exchange rate_*t*-1_				-0.000
				(0.000)
Welfare taxes (% GDP)_*t*-1_			-0.003	-0.002
			(0.003)	(0.004)
Personal inc. taxes (% GDP)_*t*-1_			-0.012***	-0.012***
			(0.003)	(0.003)
RW populist vote-share_*t*-1_				-0.002
				(0.001)
Observations	1933	1263	921	805
Countries	24	24	17	17
R^*2*^	0.989	0.989	0.991	0.992

*Note*: (1) This table portrays a pooled cross-national, time-series ordinary least squares (OLS) analysis of immigration policy in year *t*. (2) Panel-corrected standard errors are shown in parentheses. (3) Statistical significance levels:

***p < 0.001

**p < 0.01, and

*p < 0.05. (4) Country and year fixed-effects as well as country-specific time trends are included in all models.

## Outlier diagnostics: Residual-versus-fitted plot

I begin my diagnostics process by re-estimating the replicated OLS models reported in Table 1 and then use a post-estimation method, a residual-versus-fitted plot, which is the most frequently used diagnostics plot for identifying outliers. A residual-versus-fitted plot is a scatter plot of residuals on the *y*-axis and fitted values (estimated responses) on the *x*-axis. In a well-fitted OLS regression model, there should be no pattern to the residuals plotted against the fitted values [20].

**Fig 1:**
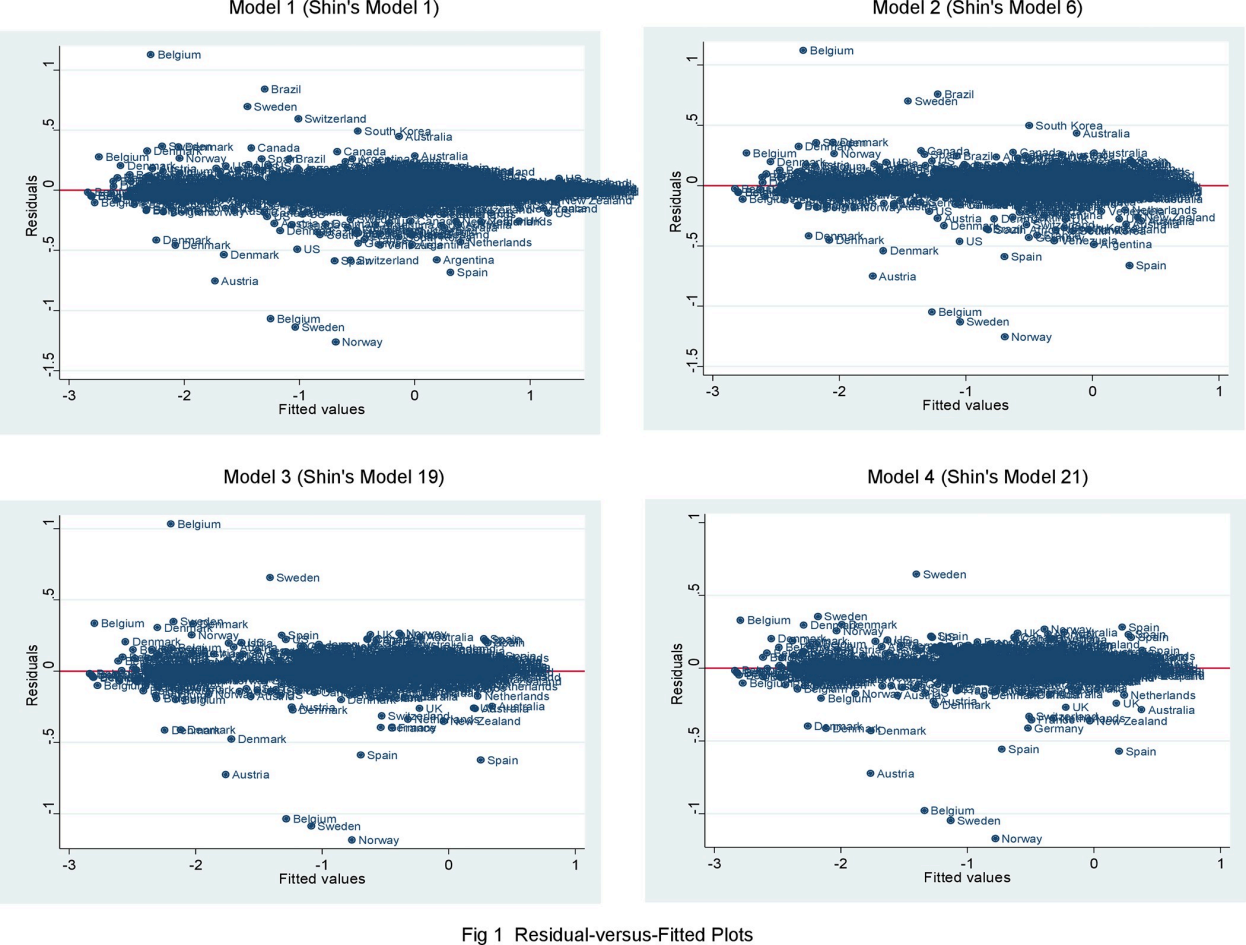
Residual-versus-fitted plots.

I re-estimate the replicated OLS Models 1 to 4 of Table 1 before drawing Fig 1 which includes four residual-versus-fitted plots. The figure displays some abnormal patterns. Several countries such as Norway and Belgium deviate significantly from the residual = 0 line, indicating that those residuals stand out from the basic random pattern of residuals. I mark them as possible outliers.

To analyze how outlying countries affect OLS results, I re-estimate the replicated OLS regression models without outlying cases. I then compare the re-estimated results with those with outlying cases. I choose Norway as an outlying case since it is the farthest one from the residual = 0 line in each of the four residual-versus-fitted plots. After dropping Norway from the sample countries, I re-estimate the replicated four models that appeared in Table 1 and report the re-estimated coefficients and standard errors in Table 2. To compare the four models, I use two information criteria of goodness of fit: AIC and BIC. The rule of thumb is that between two models, the one with the smaller AIC/BIC fits the data better than the one with the larger AIC/BIC. These statistics indicate that among the four models, Model 1 is best-fitting and Model 4 is least-fitting. These statistics underscore the importance of building a statistical model parsimoniously. Achen [20] and Ray [21] indeed advocate parsimonious models by arguing that too many control variables in regression models spoil the broth. When perusing Table 2, I notice that Ln (oil income per capita) _*t-*1_ is not significantly different from zero across the models except for the last one (significant at the 0.05 level in Model 4). Tariff rate_*t*-1_ achieves significance at the 0.05 level in only one of the two multiplicative models (Model 3). The interaction term Ln(oil income pc) × tariff_*t*-1_ does not come out as a significant and positive predictor in both models (Models 3 and 4).

**Table 2:** Oil wealth and immigration policy: Norway dropped.

* *	1	2	3	4
* *	1801–2013	1946–2013	1951–2013	1961–2012
* *	Shin’s Model 1	Shin’s Model 6	Shin’s Model 19	Shin’s Model 21
Immigration policy_*t*-1_	0.924***	0.908***	0.895***	0.874***
	(0.006)	(0.008)	(0.015)	(0.017)
Ln(oil income per capita)_*t*-1_	-0.004	-0.006	-0.012	-0.019*
	(0.002)	(0.004)	(0.009)	(0.009)
Tariff rate_*t*-1_			0.006*	0.003
			(0.003)	(0.005)
Ln(oil income pc) × tariff_*t*-1_			0.001	0.001
			(0.001)	(0.001)
Ln(GDP per capita)_*t*-1_		-0.052	-0.063	-0.094
		(0.028)	(0.046)	(0.081)
GDP growth_*t*-1_		0.080	-0.122	-0.000
		(0.073)	(0.172)	(0.210)
Ln(population)_*t*-1_		0.103	-0.144	-0.740**
		(0.112)	(0.234)	(0.285)
Polity score_*t*-1_		-0.009*	-0.008	-0.045*
		(0.004)	(0.008)	(0.018)
Real effective exchange rate_*t*-1_				0.000
				(0.000)
Welfare taxes (% GDP)_*t*-1_			-0.001	0.002
			(0.003)	(0.004)
Personal inc. taxes (% GDP)_*t*-1_			-0.012***	-0.012***
			(0.003)	(0.003)
RW populist vote-share_*t*-1_				-0.002
				(0.001)
Observations	1870	1201	861	755
Countries	23	23	16	16
R^*2*^	0.990	0.990	0.991	0.992

*Note*: (1) This table portrays a pooled cross-national, time-series ordinary least squares (OLS) analysis of immigration policy in year *t*. (2) Panel-corrected standard errors are shown in parentheses. (3) Statistical significance levels:

***p < 0.001

**p < 0.01, and

*p < 0.05. (4) Country and year fixed-effects as well as country-specific time trends are included in all models.

The diagnostic analysis performed after the removal of only one outlying country, Norway, weakens the robustness of the core findings reported in replicated Table 1 (and in Shin’s study overall). The results of the diagnostics are also troublesome for Shin’s case study of Norway because the country is not a typical oil-rich democracy, but an outlier that distorts the real relationships among oil wealth, tariff rate, and immigration policy. Note that when I drop both Norway and Belgium from the sample, as shown in S1 Appendix, the results are similar to those in Table 2. This is not surprising given that Belgium is not as influential as Norway and, thus, it minimally affects the predicted line. The diagnostic results from the residual-versus-fitted plots call for further analysis of outliers in the sample data.

## Outlier diagnostics: Leverage-versus-squared-residual plot

In this section, I employ a more advanced diagnostic graph that exhibits the leverage versus the (normalized) residuals squared. Through this visual presentation, I can observe how cases that are outliers on an independent variable have more leverage than cases that are closer to the mean of an independent variable–points above the horizontal line have a higher-than-average leverage; points to the right of the vertical line have larger-than-average residuals. A leverage-versus-squared-residual plot is one of the most useful diagnostic graphs for detecting outliers, while a residual-versus-fitted plot is the most frequently used diagnostic graph [22].

**Fig 2:**
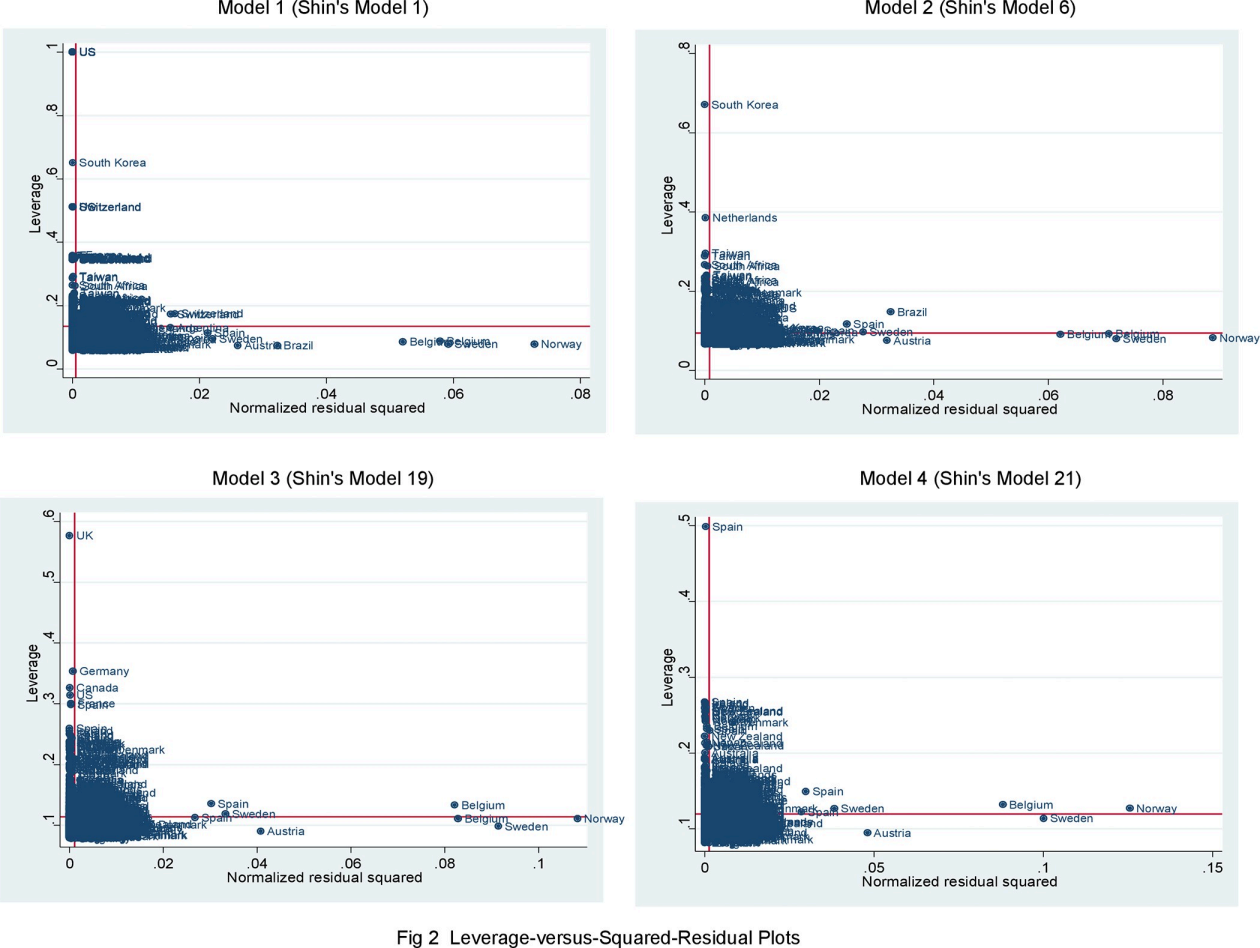
Leverage-versus-squared-residual plots.

Based on the replicated OLS Models 1 to 4 of Table 1, I draw Fig 2. Each of the four graphs in the figure presents Norway as a country with a very large residual (i.e., the difference between the predicted and observed value for Norway is exceptionally large), but with low leverage. In contrast, cases in the upper left of the graph, such as the U.S. under Model 1, South Korea under Model 2, the U.K. under Model 3, and Spain under Model 4, have very high leverage.

Having observed the irregular pattern of outliers in each of the four graphs, I decide to re-estimate the four replicated models of Table 1 after removing outlying countries. To alleviate outlier problems that are caused by the presence of Norway and the U.S. according to the first graphic analysis shown in Fig 2, I exclude the two countries from Shin’s sample data. I report the re-estimated results in Model 1 in Table 3. Ln (oil income per capita) _*t-*1_ fails to achieve significance even though the coefficient sign remains negative. When I exclude Norway and South Korea from Shin’s sample based on the second graphic analysis, I observe no statistical significance on Ln (oil income per capita) _*t-*1_ (see Model 2 of Table 3). When I leave out Norway and the U.K. according to the third graphic analysis and Norway and Spain according to the fourth and final graphic analysis, I find no interaction effect of Ln (oil income per capita)_*t-*1_ and Tariff rate_*t*-1_ in Models 3 and 4, respectively. Note that when I exclude Norway, the U.S., and Belgium together, as shown in S2 Appendix, the results are very similar to those of Table 3. Since Belgium’s influence is not as large as the other two countries, the predicted line is moved minimally. These diagnostic tests suggest that Shin’s models are very vulnerable to outlier problems.

**Table 3:** Oil wealth and immigration policy: Two countries dropped.

* *	1	2	3	4
* *	1801–2013	1946–2013	1951–2013	1961–2012
* *	Shin’s Model 1	Shin’s Model 6	Shin’s Model 19	Shin’s Model 21
Immigration policy_*t*-1_	0.920***	0.912***	0.897***	0.878***
	(0.007)	(0.008)	(0.016)	(0.017)
Ln(oil income per capita)_*t*-1_	-0.001	-0.006	-0.013	-0.014
	(0.002)	(0.004)	(0.014)	(0.008)
Tariff rate_*t*-1_			0.006	0.005
			(0.004)	(0.004)
Ln(oil income pc) × tariff_*t*-1_			0.001	0.001
			(0.001)	(0.001)
Ln(GDP per capita)_*t*-1_		-0.052	-0.038	-0.118
		(0.029)	(0.046)	(0.077)
GDP growth_*t*-1_		0.074	-0.149	-0.049
		(0.074)	(0.181)	(0.198)
Ln(population)_*t*-1_		0.081	-0.098	-0.598**
		(0.100)	(0.242)	(0.228)
Polity score_*t*-1_		-0.009**	-0.008	-0.028*
		(0.004)	(0.008)	(0.013)
Real effective exchange rate_*t*-1_				-0.000
				(0.000)
Welfare taxes (% GDP)_*t*-1_			-0.000	0.002
			(0.003)	(0.004)
Personal inc. taxes (% GDP)_*t*-1_			-0.011***	-0.009**
			(0.003)	(0.003)
RW populist vote-share_*t*-1_				-0.002
				(0.001)
Observations	1660	1178	804	720
Countries	22	22	15	15
R^*2*^	0.989	0.990	0.991	0.993

*Note*: (1) This table portrays a pooled cross-national, time-series ordinary least squares (OLS) analysis of immigration policy in year *t*. (2) Panel-corrected standard errors are shown in parentheses. (3) Statistical significance levels:

***p < 0.001

**p < 0.01, and

*p < 0.05. (4) Country and year fixed-effects as well as country-specific time trends are included in all models.

To visualize how tariff rate–a proxy measure for international trade openness–moderates the way through which an oil boom influences immigration policy (Hypothesis 2), Shin’s study uses a graphic presentation of the marginal effects of oil income at various levels of tariff rate from Models 19 and 21 (see p. 814). Shin’s study confidently asserts that the interaction effect between oil wealth and tariff rate matters for most of the sample data points.

**Fig 3:**
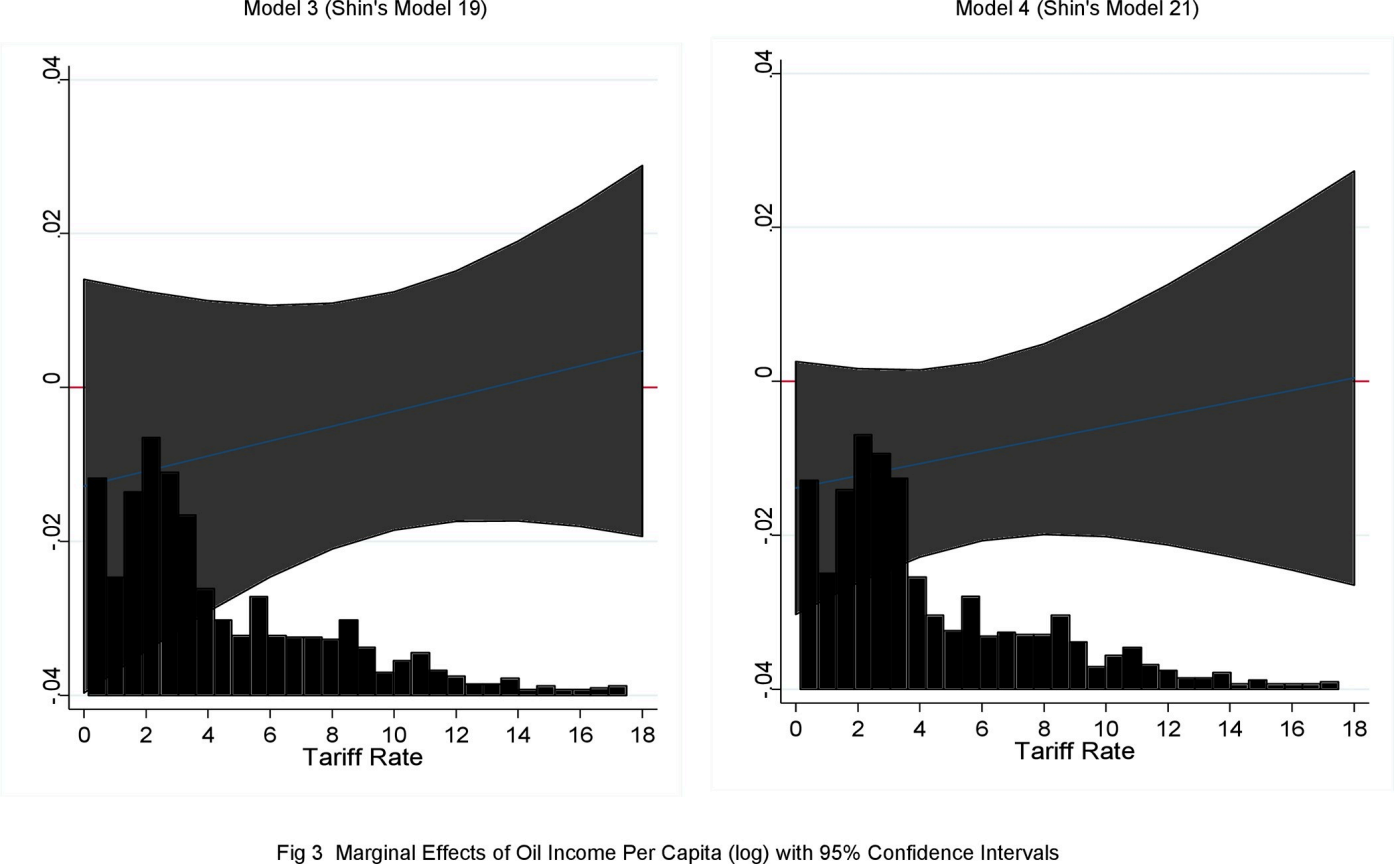
Marginal effects of oil income per capita (log) with 95% confidence intervals.

By applying the same graphic analysis of marginal effects, I draw Fig 3 after removing Norway and the U.K., and Norway and Spain, respectively. Neither Model 3 (Shin’s Model 19) nor Model 4 (Shin’s Model 21) shows any significant interaction effect in that the confidence interval includes zero along the *x* line.

To alleviate outlier problems more formally, I introduce robust regression. Some studies deem robust regression as the most effective remedial technique since it provides resistant (stable) results in the presence of outliers [13,14,17,18]. Unlike OLS regression estimation, which is inefficient and potentially biased in the presence of outliers, robust regression estimation resists the pull of outliers as it makes the latter more efficient when dealing with non-normal error distributions. Robust regression is an iteratively reweighted least-squares procedure that calculates weights. This is done for each observation using a Huber function, which down-weights observations that have larger errors and then bi-weights them from the absolute value of errors, iterating weighted least squares regression to drop the change in weights below 0.01. These Huber and bi-weight iterations are complementary to each other as the former has problems dealing with severe outliers, while the latter sometimes fails to converge or has multiple solutions.

To show the efficiency of robust regression, I report its estimated results in Table 4. Table 4 uses fewer observations than Table 1 because the Stata estimation algorithm dropped New Zealand due to collinearity. When comparing the robust regression in Table 4 with the OLS regression in Table 1, I notice that the results are fairly different, especially with respect to the coefficients of the variables of interest: Ln (oil income per capita) _*t-*1_, Tariff rate_*t*-1_, and Ln (oil income pc) × tariff_*t*-1_. None of the three key variables emerges as a significant predictor in the hypothesized direction. When the outlier problem is addressed by robust regression, I discover that oil wealth has little to do with the restrictiveness of immigration policy. In fact, Model 1 indicates even a positive, not negative, impact of oil wealth. This means that oil-rich democracies are, on average, disposed to liberalize immigration policy to attract more foreign workers.

**Table 4:** Oil wealth and immigration policy: Robust regression.

* *	1	2	3	4
* *	1801–2013	1946–2013	1951–2013	1961–2012
* *	Shin’s Model 1	Shin’s Model 6	Shin’s Model 19	Shin’s Model 21
Immigration policy_*t*-1_	1.000***	1.000***	1.000***	0.999***
	(0.001)	(0.002)	(0.003)	(0.004)
Ln(oil income per capita)_*t*-1_	0.001**	0.002	0.002	0.003
	(0.000)	(0.001)	(0.001)	(0.002)
Tariff rate_*t*-1_			-0.000	0.000
			(0.001)	(0.001)
Ln(oil income pc) × tariff_*t*-1_			0.000	-0.000
			(0.000)	(0.000)
Ln(GDP per capita)_*t*-1_		-0.010	0.007	-0.012
		(0.008)	(0.013)	(0.025)
GDP growth_*t*-1_		0.016	-0.032	-0.042
		(0.017)	(0.039)	(0.058)
Ln(population)_*t*-1_		-0.005	-0.060	-0.097
		(0.025)	(0.047)	(0.075)
Polity score_*t*-1_		0.000	0.001	-0.002
		(0.001)	(0.002)	(0.004)
Real effective exchange rate_*t*-1_				0.000*
				(0.000)
Welfare taxes (% GDP)_*t*-1_			0.001	0.001
			(0.001)	(0.001)
Personal inc. taxes (% GDP)_*t*-1_			-0.000	0.000
			(0.001)	(0.001)
RW populist vote-share_*t*-1_				0.000
				(0.000)
Observations	1914	1263	921	805

*Note*: (1) Statistical significance levels:

***p < 0.001

**p < 0.01, and

*p < 0.05. (2) Country and year fixed-effects as well as country-specific time trends are included in all models.

## Concluding remarks

By explaining how “oil booms after WWII were responsible for many democracies’ restrictive measures on low-skill immigration,” Shin’s [15 page 816] study attempts to contribute to an emerging literature on the political economy of natural resources and international migration. Yet, Shin’s empirical analysis turns out to be problematic, as it does not endure commonly used outlier diagnostics. OLS regression gives equal weight to every observation in the sample when it minimizes the residual sum of squares. However, if the sample contains outliers, every observation can not be assumed to have an equal impact on the regression results. Large outliers pull more weight than average observations [19]. When I re-examine Shin’s sample data, I discover that several outliers were not properly taken into account. After tackling the outlier problems by either dropping them from the sample data such as Norway or using robust regression that is immune to a major outlier effect, I come to the conclusion that oil-rich democracies are not associated with more strict immigration policy, regardless of their trade openness.

This null finding refutes Shin’s contention that when oil-rich democracies move away from labor-intensive industries to capital-intensive industries, they become less reliant on migrant labor, making them more prone to restrict immigration policy. Although a few countries such as Norway may fit into Shin’s theoretical explanations, I believe that many democracies digress from it due to group conflicts between pro-immigration and anti-immigration supporters [23,24]. When the national economy needs restructuring from labor-intensive to capital-intensive industries, it is difficult for democracies to reach a consensus on how to deal with immigrant workers who may or may not grease the wheels of the labor market by flowing into industries and areas where the labor shortage is a significant problem. Unlike autocratic countries, democracies must respect policy negotiations between pro-immigration groups that used to benefit from labor-intensive industries and anti-immigration groups that strive to secure their new economic fortune. This means that a democratic dialogue between old guards and new arrivals is essential, but the path forward is not without obstacles, as exemplified by immigration debates in the U.S. In fact, policy negotiations are often bogged down in the middle of the way, so many democracies are unable to make a swift and effective decision regarding restrictions on immigration. Norway was an exception to this kind of group conflict as it was able to quickly move on the industrial restructurization. Ironically, Shin (2019, 815) himself suggests this point when quoting Grytten [16]: “Norway saw deindustrialization *at a more rapid pace* than most of her largest trading partners” (emphasis added).

Overall, this replication demonstrates the significance of outlier problems in OLS regression analysis. In particular, this replication shows how the presence of outliers in Shin’s sample data led to misleading inferences and conclusions about the effects of oil wealth and tariff rates on immigration policy; therefore, outlying cases should be treated with caution. Ironically, even though Shin’s case study on Norway is subjected to an outlier problem in a statistical sense, that country has, unlike many other countries rich in raw materials, become one of the most prosperous economies through oil export [16]. Accordingly, the Norway case offers an important policy implication: if democratic countries could pull the strings of old guards and new arrivals in the process of economic restructuring in general and in the reform of immigration policy in particular, they could successfully emulate the Norwegian model of economic prosperity. Otherwise, they may not find their way out of an economic maze in which many democratic polities inadvertently enter during the complex negotiation process [25].

Future research may focus on other issues regarding Shin’s modeling strategy. To demonstrate that Shin’s finding on oil wealth and migration policy restriction is faulty, future research may (a) estimate Shin’s model in first differences, examining how changes in oil income affect changes in immigration policy; (b) refine immigration policy reforms, liberalizations, or restrictions using a binary indicator (e.g., reforms could be defined as a 1 standard deviation change/increase/decrease in score); and (c) replicate Shin’s model using other data on migration policy such as the Immigration Policies in Comparison (IMPIC) project and the Migrant Integration Policy Index (MIPEX).

## Supporting information

S1 Appendix Oil wealth and immigration policy: Norway and Belgium dropped. (DOC)

S2 Appendix Oil wealth and immigration policy: Three countries dropped. (DOC)
