# Supplementary material for: Outlier analysis: Natural resources and immigration policy
Source: PLoS One. 2022 Jan 13;17(1):e0261533. doi: 10.1371/journal.pone.0261533 (PMC8758109; doi:10.1371/journal.pone.0261533)
Supplement: S2 Appendix — (DOC) [file pone.0261533.s002.doc]

| Appendix 2 Oil Wealth and Immigration Policy: Three Countries dropped | | | | |
| --- | --- | --- | --- | --- |
|  |  |  |  |  |
|  | 1 | 2 | 3 | 4 |
|  | 1801–2013 | 1946–2013 | 1951–2013 | 1961–2012 |
|  | Shin's Model 1 | Shin's Model 6 | Shin's Model 19 | Shin's Model 21 |
| Immigration policy*_t_*_-1_ | 0.926*** | 0.919*** | 0.900*** | 0.870*** |
|  | (0.008) | (0.008) | (0.018) | (0.018) |
| Ln(oil income per capita)*_t_*_-1_ | 0.000 | -0.004 | -0.010 | -0.012 |
|  | (0.002) | (0.003) | (0.014) | (0.008) |
| Tariff rate*_t_*_-1_ |  |  | 0.000 | -0.005 |
|  |  |  | (0.005) | (0.005) |
| Ln(oil income pc) × tariff*_t_*_-1_ |  |  | 0.002 | 0.002 |
|  |  |  | (0.001) | (0.001) |
| Ln(GDP per capita)*_t_*_-1_ |  | -0.054 | -0.034 | -0.204* |
|  |  | (0.029) | (0.047) | (0.082) |
| GDP growth*_t_*_-1_ |  | 0.079 | -0.102 | 0.006 |
|  |  | (0.074) | (0.184) | (0.203) |
| Ln(population)*_t_*_-1_ |  | 0.094 | 0.009 | -0.368 |
|  |  | (0.098) | (0.252) | (0.239) |
| Polity score*_t_*_-1_ |  | -0.009* | -0.009 | -0.031* |
|  |  | (0.004) | (0.008) | (0.013) |
| Real effective exchange rate*_t_*_-1_ |  |  |  | -0.000 |
|  |  |  |  | (0.000) |
| Welfare taxes (% GDP)*_t_*_-1_ |  |  | 0.002 | 0.003 |
|  |  |  | (0.003) | (0.004) |
| Personal inc. taxes (% GDP)*_t_*_-1_ |  |  | -0.015*** | -0.016*** |
|  |  |  | (0.003) | (0.003) |
| RW populist vote-share*_t_*_-1_ |  |  |  | -0.001 |
|  |  |  |  | (0.001) |
| Observations | 1597 | 1116 | 744 | 668 |
| Countries | 21 | 21 | 14 | 14 |
| R*^2^* | 0.988 | 0.990 | 0.992 | 0.993 |
| *Note*: (1) This table portrays a pooled cross-national, time-series ordinary least squares (OLS) analysis of immigration policy in year *t*. (2) Panel-corrected standard errors are shown in parentheses. (3) Statistical significance levels: ****p* < 0.001, ***p* < 0.01, and **p* < 0.05. (4) Country and year fixed-effects as well as country-specific time trends are included in all models. | | | | |
